# Diagnostic utility of musculoskeletal ultrasound in patients with suspected arthritis – a probabilistic approach

**DOI:** 10.1186/s13075-014-0448-6

**Published:** 2014-10-01

**Authors:** Hamed Rezaei, Søren Torp-Pedersen, Erik af Klint, Magnus Backheden, Yogan Kisten, Noémi Györi, Ronald F van Vollenhoven

**Affiliations:** Unit of Clinical Therapy Research, Inflammatory Diseases (ClinTRID), Karolinska Institute, Stockholm, Sweden; Department of Rheumatology, Karolinska University Hospital, Stockholm, Sweden; Department of Diagnostics, University of Copenhagen Hospital, Glostrup, Denmark; Department of Learning, Informatics, Management and Ethics (LIME), Karolinska Institute, Stockholm, Sweden

## Abstract

**Introduction:**

This study aimed to assess the utility of musculoskeletal ultrasound (MSUS) in patients with joint symptoms using a probabilistic approach.

**Methods:**

One hundred and three patients without prior rheumatologic diagnosis and referred to our clinic for evaluation of inflammatory arthritis were included. Patients were assessed clinically including joint examination, laboratory testing including acute-phase reactants, rheumatoid factor (RF) and anti citrulinated protein antibody (ACPA), and radiographs of hands and feet if clinically indicated. A diagnostic assessment was then performed by the responsible rheumatologist where the probability of a) any inflammatory arthritis and b) rheumatoid arthritis was given on a 5-point scale ranging from 0 to 20% up to 80 to 100% probability. Subsequently, an ultrasound examination of the wrist, metacarpophalangeal (MCP), proximal interphalangeal (PIP) joints 2 to 5 in both hands, metatarsophalangeal (MTP) joints 2 to 5 in both feet and any symptomatic joints was performed and the results presented to the same rheumatologist. The latter then assessed the diagnostic probabilities again, using the same scale.

**Results:**

The rheumatologists’ certainty for presence/absence of inflammatory arthritis and rheumatoid arthritis was increased significantly following ultrasound performance. The proportion of patient for whom diagnostic certainty for inflammatory arthritis was maximal was 33.0% before and 71.8% after musculoskeletal ultrasound (*P* <0.001). With regard to a diagnosis of RA, the proportions were 31.1% pre-test and 61.2% post-test (*P* <0.001). MSUS findings agreed with the final diagnosis in 95% of patients.

**Conclusion:**

Musculoskeletal ultrasound, when added to routine rheumatologic investigation, greatly increases the diagnostic certainty in patients referred for the evaluation of inflammatory arthritis. The changes from pre-test to post-test probability quantify the diagnostic utility of musculoskeletal ultrasound in probabilistic terms.

## Introduction

Musculoskeletal complaints are exceedingly common in the population and a large proportion of patients with severe, refractory, or unclear joint symptoms are referred to rheumatology units for further diagnostic evaluation. The traditional evaluation of patients with joint symptoms primarily includes medical history and physical examination, complemented by blood tests including rheumatoid factor (RF) and anti-citrullinated protein antibody (ACPA), synovial fluid examination, and radiography of the affected joints [1,2]. Although the traditional methods are well-established, there are still a sizeable proportion of patients in this category who are not reliably diagnosed in the early stages of the disease [3].

Musculoskeletal ultrasound (MSUS) is a reliable, cost effective, patient-friendly and safe imaging modality used as a complement to other diagnostic methods in rheumatology [4-6]. MSUS has been shown to be superior to clinical examination to identify synovitis [7-10] and according to the European League Against Rheumatism (EULAR) recommendation for the use of imaging in the clinical management of rheumatoid arthritis (RA), when there is diagnostic doubt, MSUS can be used to improve the certainty of diagnosis above clinical signs and symptoms alone [11]. Although gray-scale MSUS does have an important role to play in synovitis identification, color or power Doppler imaging are more beneficial in identifying active inflammation by detecting hypervascularisation and hyperemia in synovial inflammation [12].

Quantitative analyses of the diagnostic utility of MSUS in patients with arthritis in rheumatologic practice have been done in smaller groups of patients [3,13-15]. A study by Matsos *et al*. showed that synovitis in the hands and feet detected by MSUS improved the certainty of a diagnosis of seronegative arthritis and significantly influenced the rheumatologist’s confidence in the management plan [15]. Freeston *et al*. reported that in patients with arthritis of unknown etiology, combining power Doppler ultrasound (PDUS) with conventional assessment can help rheumatologists to achieve a higher certainty of diagnosis [3]. In contrast, a recent study based on a retrospective analysis of clinical datasets from an early arthritis cohort in the UK showed that MSUS provided no additional discriminatory value to predict persistent inflammatory arthritis [16]. Nonetheless, EULAR has recommended the use of MSUS in this setting [11].

To define the diagnostic utility of a test, two fundamentally different approaches can be taken. In a classical (deterministic) analysis, the performance of the test is measured against a gold standard, leading to value for sensitivity, specificity, and positive and negative predictive value. This type of analysis makes the underlying assumption that a diagnosis is either present or absent, that it is an all-or-none phenomenon. In clinical practice, this is not necessarily the case, and clinicians assessing patients with early arthritis often have to express their diagnosis as probabilities. Therefore, in the other alternative, a probabilistic (Bayesian) analysis, one accepts that there is a range of diagnostic uncertainties or certainties, from highly unlikely to highly likely, and assesses the degree to which the addition of a test changes the diagnostic certainty. This study aimed to assess the diagnostic impact of MSUS findings in patients referred for rheumatologic evaluation because of suspected inflammatory arthritis primarily using a probabilistic approach.

## Methods

### Patients and clinical assessment

Patients in this study were recruited consecutively between 2010 and 2013. All patients had suspected inflammatory arthritis but had no prior rheumatologic diagnosis. They reported inflammatory symptoms, mostly from the hands and feet, such as arthralgia, stiffness and swollen joints. All patients had been referred by general practitioners to the early arthritis clinic. As is always done, a first clinical assessment was performed by a rheumatologist, based on medical history, physical examination, and review of previously performed laboratory and/or radiological studies. The assessment was usually complemented by new blood tests including anti-citrullinated protein antibody (ACPA), rheumatoid factor (RF) and/or acute phase reactants. Radiographic assessment of the hands and feet was also performed. No MSUS assessment was done at this time point. The patients were invited to participate in the study and after informed consent was given, the rheumatologist completed the study case-report form (CRF) which included her/his assessment of the likelihood that the patient had: a) inflammatory arthritis; b) RA. In this pre-test assessment, the physician based likelihood on all available clinical and laboratory data but without any MSUS information on a five-point scale: very likely (≥80%), likely (≥60% and <80%), possible (≥40% and <60%), not likely but possible (≥20% and <40%) and very unlikely (<20%).

Importantly, in some analyses we considered patients with the highest and lowest degrees of diagnostic likelihood as one group, representing the group of highest diagnostic certainty. Thus, we included those patients where the diagnosis was ≥80% likely and those where the diagnosis was <20% likely, because both of these groups represented a high degree of confidence on the part of the clinician regarding the diagnosis, contrasting with those patients where the diagnosis was 40% to 60% likely and who represented the greatest diagnostic uncertainty.

Subsequently MSUS was performed by one sono-rheumatologist (HR) and the results, given descriptively as morphological and vascularization data of the studied joints were presented for post-test assessment to the same rheumatologist who had performed the pre-test evaluation. The latter then assessed the diagnostic probabilities again, using the same five-point scale. In the present study, there were four rheumatologists in total who met the patients from the beginning and performed the pre-test and post-test assessment on a five-point scale. As an additional control, the participating patients’ cases were described as vignettes, once without and once with the results of the MSUS examination. These vignettes were then scored by another rheumatologist in random order, again using a probabilistic approach.

We also analyzed the utility of MSUS in classical terms, based on the final diagnosis that was obtained for each patient by the same rheumatologist who did the pre-test and post-test evaluation. The predictive accuracy of MSUS was assessed when every patient had final diagnosis at the end of the follow-up time that was one year after inclusion of the last patient. In general, RA was diagnosed if 1987 American College of Rheumatology (ACR) classification criteria [17] or 2010 ACR/EULAR classification criteria [1] were fulfilled. Psoriatic arthritis (PsA) was diagnosed based on clinical judgment, performed by the rheumatologist and not necessarily based on classification criteria. Undifferentiated inflammatory arthritis (UIA) was diagnosed when arthritis was confirmed but classification criteria for a specific rheumatologic disease were not fulfilled. These diagnoses were re-analyzed one year after inclusion of the last patient. We also studied the treatments that the patients had been given during a follow up of 1 to 4 years. This study was approved by Regional Ethical Review Board in Stockholm. All participants gave written informed consent before inclusion.

### Musculoskeletal ultrasound assessment

**Figure 1:**
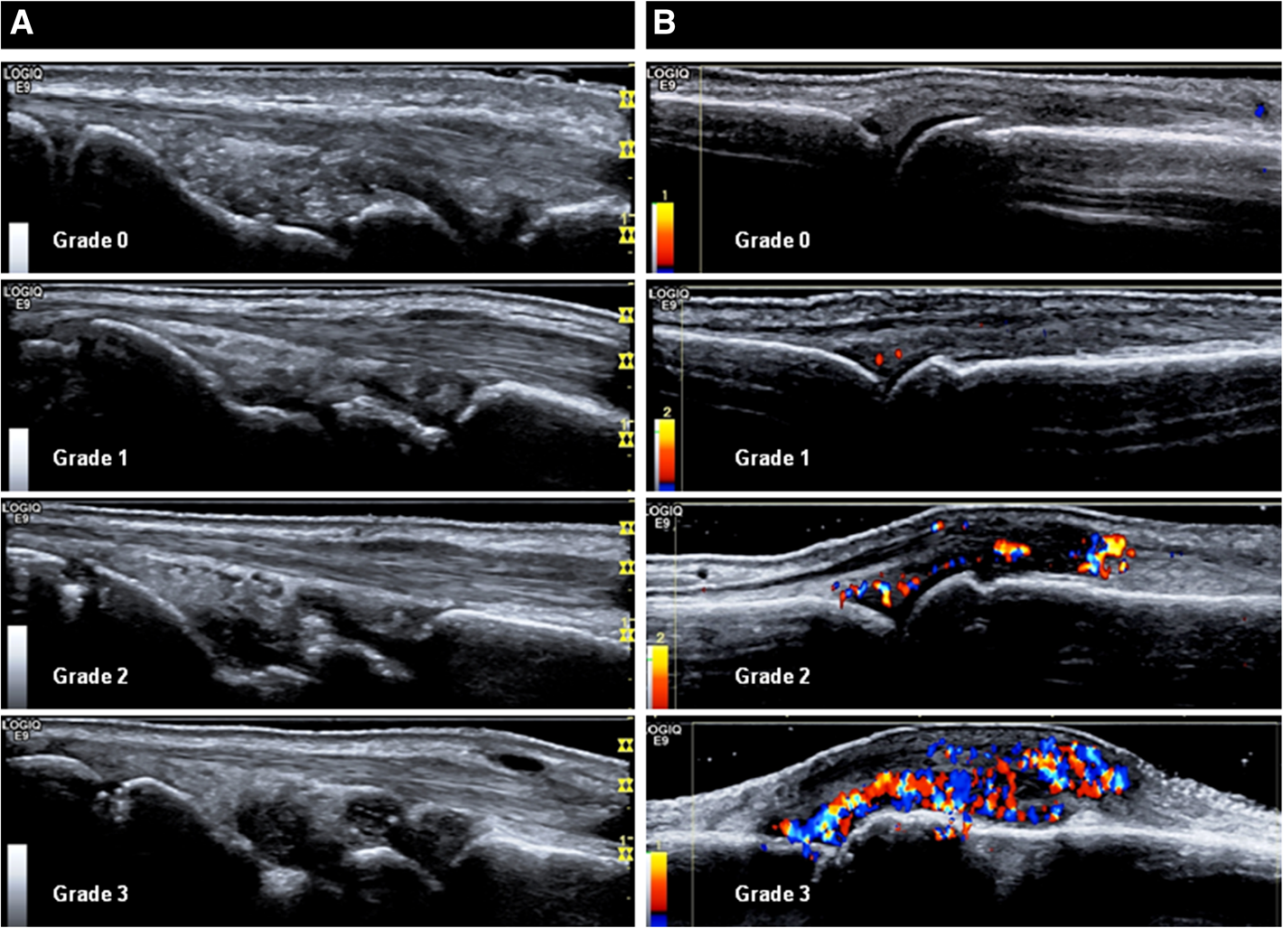
**Semiquantitative ultrasound score in B-mode at the wrist joint in the central dorsal longitudinal position (A) and color Doppler semiquantitative ultrasound at the metacarpophalangeal (MCP) joints in the dorsal longitudinal position (B).** Cut off for positive musculoskeletal ultrasound finding was grade one in both B-mode and color Doppler ultrasound in this study.

MSUS, including B-mode and color Doppler ultrasound (CDUS) was performed by one sono-rheumatologist (HR), who had 6 years experience of performing/reading MSUS. The MSUS findings were presented to the four rheumatologists as a report in the patient’s journal, of joints and/or tendons with thickened synovium with or without Doppler activity. The findings were interpreted as active synovitis/tenosynovitis (Doppler-positive) or signs of prior synovitis/tenosynovitis (Doppler-negative thickening) in four categories: arthritis certain (one or more joints with hyperemia in synovial hypertrophy); arthritis very likely (two or more joints with Doppler-negative synovial hypertrophy or two with tenosynovitis or one Doppler-negative synovial hypertrophy and one with tenosynovitis); arthritis possible (one joint with Doppler-negative synovial hypertrophy or one with tenosynovitis) and arthritis unlikely (normal ultrasound findings). The categorization was only performed in order to investigate the reliabiliy of MSUS evaluation performed by the sono-rheumatologist. We compared the MSUS findings with the final diagnosis and the number of patients with anti-rheumatic treatment at the end of the follow-up time. MSUS findings were categorized as positive or negative findings in B-mode and CDUS and the cut off was grade 1 for definition of positive signs in both B-mode and CDUS (Figure 1) according to the scoring system by Ohrndorf *et al*. [18]. The General Electric LOGIQ E9 unit (Wauwatosa, WI, USA) with a linear array transducer was used for this study. The B-mode frequency was 15 MHz. The CDUS setting was as follows: frequency of 10 MHz, pulse repetition frequency of 0.5 KHz, and wall filter of 69 Hz. The MSUS examination was divided in two parts: a) mandatory joints b) symptomatic joints. Bilateral wrist (including radiocarpal and midcarpal), metacarpophalangeal (MCP) joints, proximal interphalangeal (PIP) joints, flexor tendons in the second to fifth fingers and metatarsophalangeal (MTP) joints 2 to 5 in the feet were scanned in all the patients as mandatory joints. Scanning of the wrist included central, radial and ulnar dorsal longitudinal positions. Volar scanning of the wrist was performed if there was suspected flexor tenosynovitis in this area. MCP and MTP joints 2 to 5 were scanned with dorsal and PIP joints 2 to 5 with the volar longitudinal position. For symptomatic joints, if the patients reported symptoms from joints other than the above, the sono-rheumatologist scanned these symptomatic joints at the same time in order to see if there were any sonographic changes for synovitis, tenosynovitis, tendinitis/enthesitis or bursitis in these areas, both in B-mode and CDUS.

### Statistical analysis

Normally distributed measures were presented as mean (SD) group differences and were analyzed with Student’s *t*-test. Changes in proportions from pre-test to post-test were analyzed by the McNemar test. Differences in proportions between groups were analyzed with Fisher’s exact test or the chi-square test. A marginal homogeneity model for repeated measures was used for analyzing the change in diagnosis in association with ultrasound findings.

## Results

### Patient characteristics and ultimate diagnosis

One hundred and three patients with a mean age (SD) of 50 (16.4) years were included consecutively in this study. The proportion of patients with ACPA and RF positivity was 29% and 34%, respectively. The mean (SD) symptom duration was 8.5 (3.8) months: 76 patients (73.8%) were female.

At the end of the follow-up time, 65% (67/103) of patients were diagnosed as having any inflammatory arthritis, while the remainder did not receive a specific rheumatologic diagnosis: 36.9% (38/103) of patients were diagnosed as having RA; 21.4% (22/103) had UIA; 5 patients were diagnosed with PsA, one with polymyositis, and one with gout. Patients who were found to have no rheumatologic disease were referred back to the general practitioner; a review of data in the Swedish Rheumatology Quality registry (SRQ) at the end of the follow-up time showed that no one in this group had been referred to the rheumatology departments again during the study period. At the end of follow up, 53.4% of the original cohort (55/103) had been given disease-modifying anti-rheumatic drugs (DMARDs) of whom 35 were treated with methotrexate (MTX) and 14 had other DMARDs. Eighteen patients were treated with biologics as monotherapy or in combination with DMARDs.

### Impact of MSUS on diagnostic likelihood at first assessment of early arthritis

**Figure 2:**
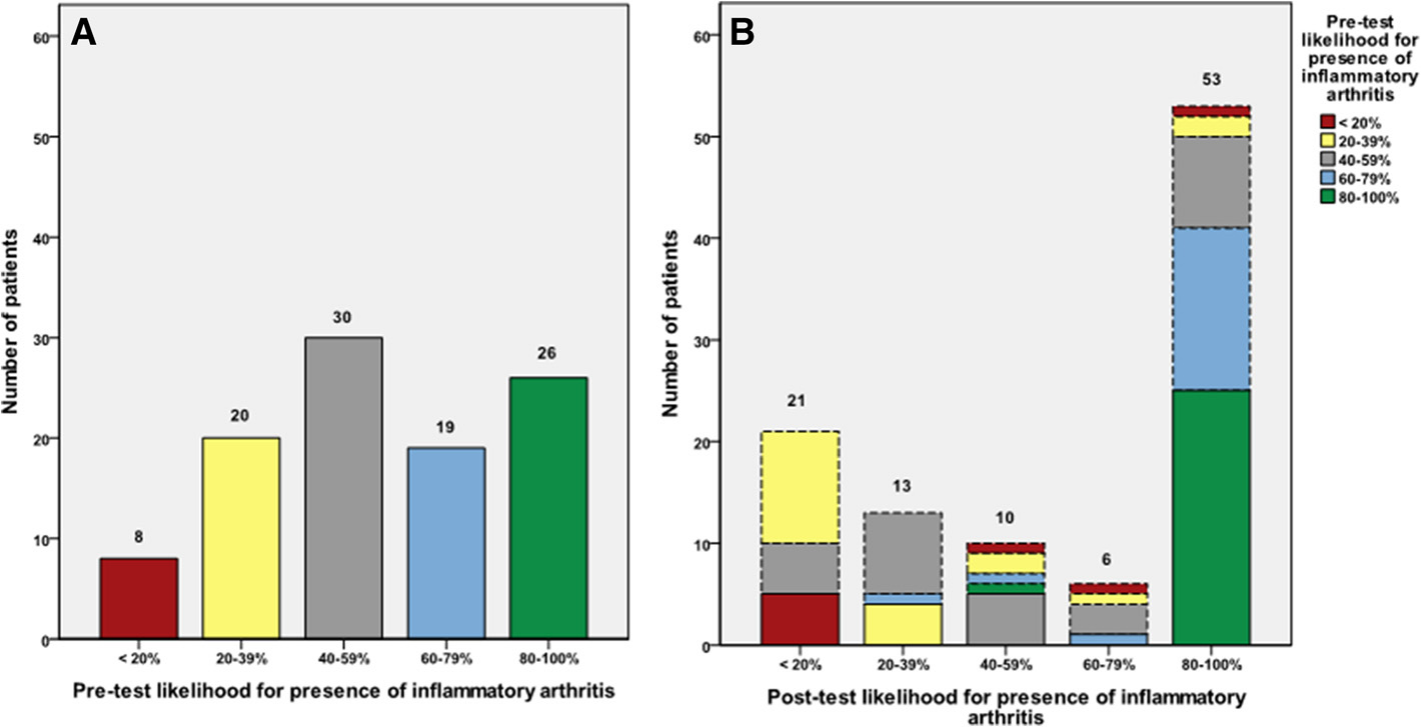
**Likelihood for inflammatory arthritis.** **(A)** Pre-test and **(B)** post-test likelihood for presence of inflammatory arthritis.

MSUS assessment showed that 63.1% (65/103) of patients had ultrasound findings in B-mode and 58.3% (60/103) in CDUS, indicating inflammatory arthritis. With regard to the clinical likelihood of having (any) inflammatory arthritis, the proportion of patients for whom diagnostic certainty was maximal (those with <20% and ≥80% likelihood) was 34/103 (33.0%) before MSUS and 74/103 (71.8%) after MSUS (McNemar test *P*-value <0.001). With regard to the probability of RA, the proportions were 32/103 (31.1%) pre-MSUS and 63/103 (61.2%) post-MSUS (McNemar test *P* -value <0.001). Parallel reductions were seen in the proportions of patients with greatest diagnostic uncertainty (40% to 60% likelihood), from 30/103 (29.1%) to 10/103 (9.7%) (McNemar test *P*-value = 0.06) for diagnosis of (any) inflammatory joint disease and from 26/103 (25.2%) to 8/103 (7.8%) (McNemar test *P*-value = 0.08) for diagnosis of RA, respectively. Figure 2 demonstrates the impact of MSUS information when it was presented to the rheumatologist for post-test assessment showing how the two groups with the highest and lowest likelihood score increased and how the middle group decreased. As shown, 27 patients moved toward lesser likelihood for inflammatory arthritis and 39 moved toward higher likelihood. The logistic linear model also showed a significant association between MSUS findings and change in diagnostic certainty for any inflammatory arthritis and for RA (*P* <0.0001). When the cases were re-scored by another independent rheumatologist based on case vignettes without and with MSUS, nearly identical results were obtained as in the original scoring (data not shown).

### Relationship between MSUS at first evaluation and ultimate diagnosis

**Figure 3:**
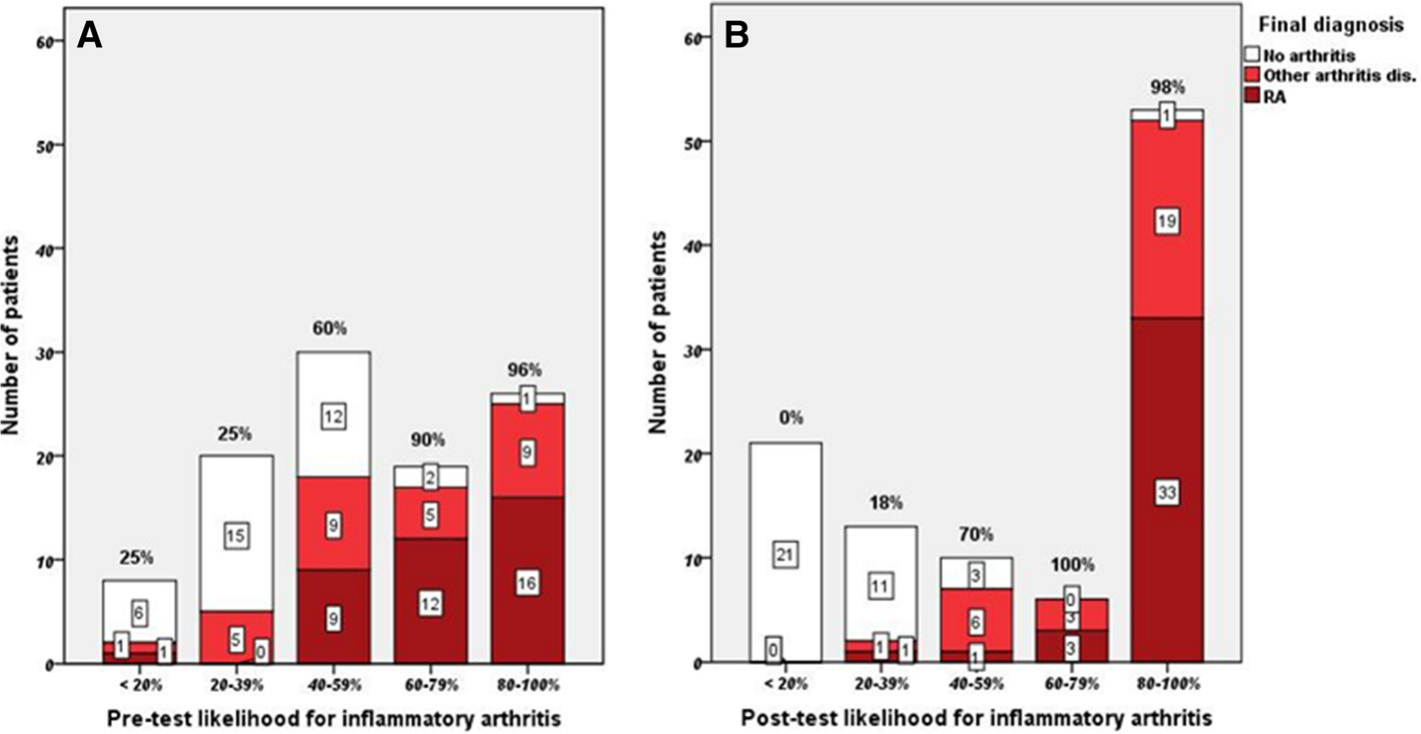
**Proportion of patients with a different final diagnosis in pre-test (A) and post-test (B) evaluation of likelihood of inflammatory arthritis.** The percentages above the columns show the fraction of inflammatory arthritis within each column. RA, rheumatoid arthritis.

Overall, of the patients who were included in the study 65% were diagnosed with any inflammatory arthritis and 36.9% with RA. Figure 3 demonstrates the final diagnosis in the groups categorized by pre-test and post-test likelihoods and how these initial risks changed within the groups to higher or lower risks in post-test evaluation. As shown, the rheumatologists’ pre-MSUS evaluation was significantly less accurate when compared to the final diagnosis, and was particularly wanting in being able to identify with confidence those patients without inflammatory arthritis.

**Figure 4:**
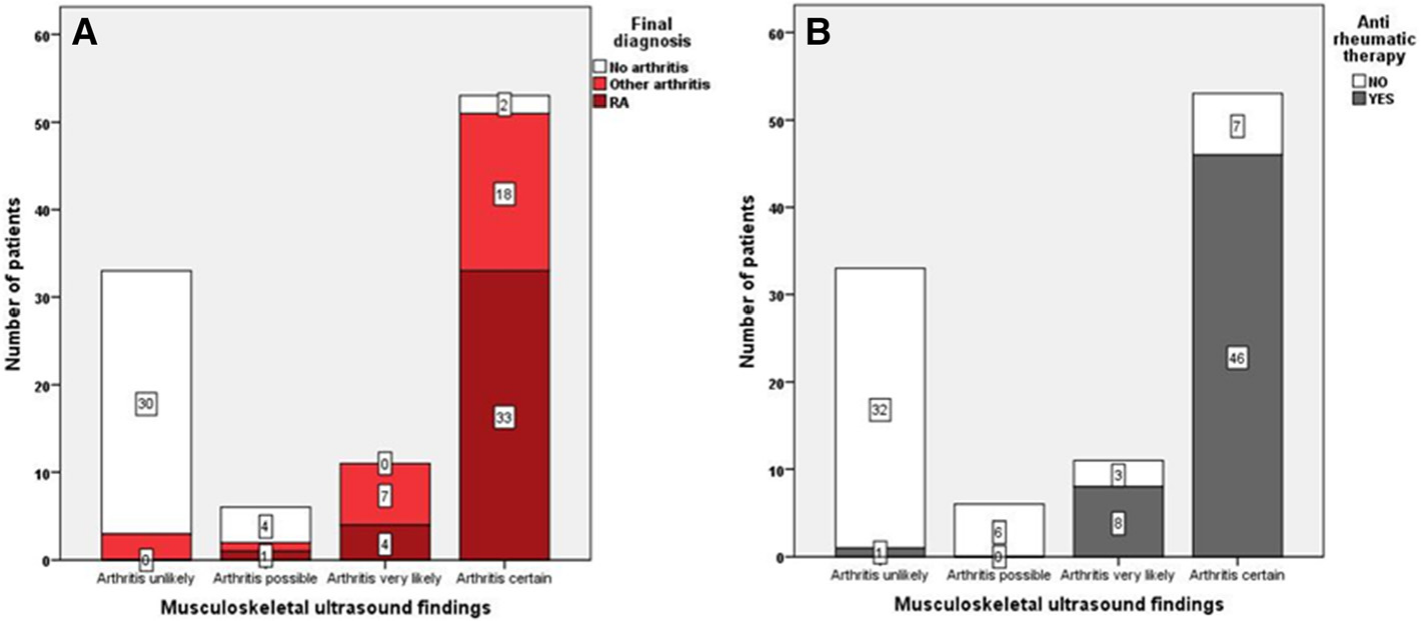
**Proportion of patients with three final diagnosis (A) and ongoing anti-rheumatic treatment at the end of the follow up time (B) in four categories with different degrees of musculoskeletal ultrasound diagnostic certainty.** RA, rheumatoid arthritis.

**Figure 5:**
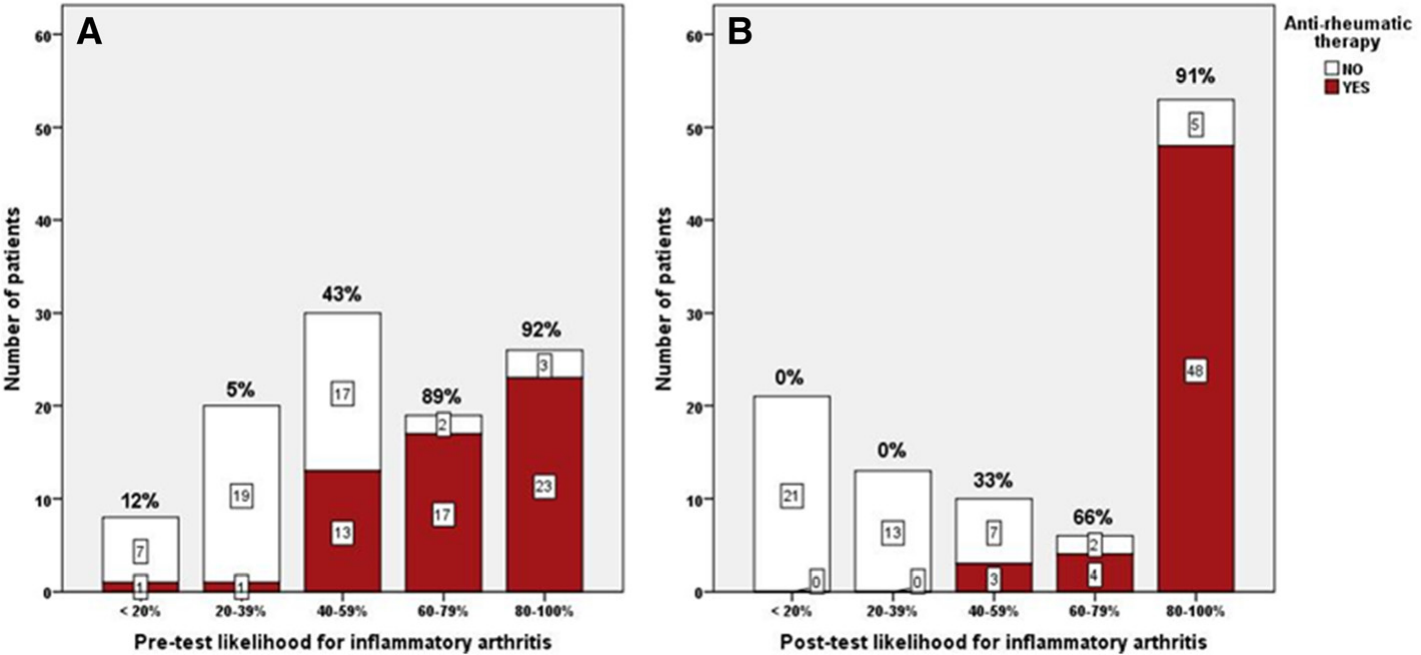
**Proportion of patients on anti-rheumatic therapy, including DMARDs, corticosteroids and biologics in pre-test (A) and post-test (B) evaluation of likelihood for inflammatory arthritis.** RA, rheumatoid arthritis.

With regard to the final rheumatologic diagnosis and accuracy of MSUS to establish the diagnosis inflammatory arthritis or not, based on positive and negative findings, Figure 4A demonstrates the impact of MSUS on both confirming and denying the presence of arthritis. In the vast majority (>95%) of patients, there was agreement between the MSUS findings and final diagnosis. A similar result was obtained when the accuracy of MSUS was investigated in terms of the number of patients on anti-rheumatic treatment at the end of the follow-up time, as shown in Figure 4B. The proportion of patients for whom diagnostic certainty was more than 80% and who were being treated with anti-rheumatic therapy (DMARDs, biologics and corticosteroids) was 23/103 (22.0%) before MSUS and 48/103 (46.6%) after MSUS (McNemar test *P*-value <0.001). As shown in Figure 5B, when the post-test likelihood for inflammatory arthritis was below 40% there was no prescription of anti-rheumatic treatment, which demonstrated the practical consequence of using MSUS. However when the pre-test diagnostic certainty was below 40%, the patients were very unlikely to be prescribed anti-rheumatic therapy (2 of 28 patients, as shown in Figure 5A). The data for anti-rheumatic treatment were also checked in the patients’ journals one year after inclusion of the last patient. The decision to treat patients with anti-rheumatic therapy, including DMARDs, biologics and corticosteroids, was made after knowing the ultrasound results.

## Discussion

In this study, we addressed the diagnostic utility and benefit of MSUS when added to the conventional rheumatologic diagnostic assessment in patients with suspected inflammatory arthritis, using a probabilistic approach. Thus, the core of the paper was the difference between pre-test and post-test assessments of diagnostic uncertainty or certainty, and we demonstrated a marked impact of MSUS findings upon the rheumatologist’s evaluation of likelihood of any inflammatory arthritis in general and for RA specifically. The results also indicate that our rheumatologists have learned to use MSUS in the diagnosis of early inflammatory arthritis. We found that MSUS greatly increased the diagnostic certainty for diagnosis of inflammatory arthritis in general and for RA in particular, which is consistent with a previous study by Matsos *et al*. [15]. Moreover MSUS increased both the positive diagnostic certainty (≥80% certain of diagnosis) and the negative diagnostic certainty (<20% likely to have the diagnosis), while greatly reducing the number of patients where diagnostic uncertainty was maximal. These findings provide quantitative support for the utility of MSUS in the evaluation of patients with suspected arthritis when there is diagnostic doubt, as also supported by the EULAR recommendation for the use of imaging of the joints in the management of RA [11]. As expected, among patients with early arthritis symptoms the likelihood of having any inflammatory arthritis, and especially of having RA, increased with the presence of MSUS findings. In addition, we found that MSUS also improved diagnostic accuracy compared to clinical assessment alone, when analyzed in a classical (deterministic) manner; as also shown previously in >95% of patients there was agreement between MSUS findings and the final diagnosis.

In the study by Matsos *et al*. [15], 62 patients were referred by two rheumatologists for MSUS scanning of the hands and feet and the diagnostic confidence for diagnosis was made before and after MSUS evaluation. In that study, the rheumatologist’s certainty for seronegative arthritis was significantly increased (46.8% versus 61.3%, *P* = 0.05) but no significant increase in diagnostic certainty for RA was observed (46.8% versus 61.3%, *P* = 0.17). Our results for increased diagnostic certainty of RA were highly significant (31.1% versus 61.2%, *P* <0.001). McNemar's test was performed in both studies to determine differences in pre-test test and post-test diagnostic probability. In the study by Matsos *et al*., only joints requested by the rheumatologists were scanned rather than a pre-specified number of joints in the hands and feet as in our study.

Freeston *et al*. analyzed the predictive value of MSUS in the diagnosis of inflammatory arthritis in 50 ACPA- and RF-negative patients with significant diagnostic uncertainty. In that study, positive MSUS findings increased the probability of inflammatory arthritis from between 2% and 30% to between 50% and 94% [3]. In the present study, we observed that MSUS results (both positive and negative findings) raised the diagnostic certainty from 33% to 71.8% for any inflammatory arthritis. Theoretically this might have less to do with the patient’s final diagnosis and more with the rheumatologist’s certainty on post-test evaluation. However, as shown in Figure 3, the diagnosis of patients with inflammatory arthritis in general and RA specifically as the final diagnosis, moved to greater certainty on post-test evaluation. In our study, increase in diagnostic certainty was observed in both ACPA/RF- positive and -negative patients.

A recent retrospective study by Pratt *et al*. showed that MSUS as a routine supplement in early arthritis patients did not add any substantial discriminatory value for predicting persistent inflammatory arthritis. Among 379 patients, seven clinical and serological variables had independent and significant associations with persistent arthritis. The risk metric for clinical and serological variables was shown as having excellent discriminatory ability (area under the curve = 0.91, *P* <0.001). Addition of MSUS did not further improve predictive accuracy and the diagnostic utility of the new metric was equivalent to the previous one [16]. Our study showed that in most of the patients MSUS had an excellent association with higher diagnostic certainty. One key difference between the two studies is the number of scanned joints, which was 16 in that study and at least 26 in our study. We performed MSUS of the wrist in three positions (as described in Methods) while scanning of the wrist was not performed in that study. Prior studies showed the sensitivity value for scanning of the wrist in inflammatory arthritis [14,19]. Another difference here is that we had a prospective design and our focus was on whether MSUS could influence and increase the diagnostic certainty during the rheumatologic investigation. Our focus in this study was not to evaluate the sensitivity and specificity of MSUS in early arthritis as the validity of MSUS for detecting synovitis has been confirmed by several previous studies [4,7,9,10,20].

A study by Kelly *et al*. presented as an abstract at the EULAR congress 2013 [21] showed that routine use of MSUS for patients with suspected inflammatory arthritis was associated with earlier diagnosis and earlier initiation of therapy in patients with RA as the final diagnosis. In that study, the patients were divided into two groups, those who were diagnosed by MSUS versus those who were not. A significantly greater proportion of patients in the MSUS group received a final diagnosis at their first visit and a similar difference was observed for patients with a diagnosis of RA. Where patients had a diagnosis of RA, there was a significant difference in the time to diagnosis and time to initiation of therapy. The main difference between our study and that study is that we performed MSUS for all patients after the first visit. Our study was not designed to show a difference between patients having MSUS and those not having it. However our result is consistent with that study and as shown in our results, most of the patients with positive MSUS finding had anti-rheumatic therapy at the end of the follow-up time.

Our study has limitations and perhaps the main one was that the treating rheumatologist who performed the scoring of diagnostic certainty/uncertainty after MSUS was aware of her/his own scoring before MSUS and may have felt motivated to improve the result. Moreover, pre-test assessment was already done under the assumption that more information (in this case MSUS results) would be available at a later stage, which maybe lead to increased post-test probability. However, when the cases were re-scored by another independent rheumatologist on the basis of vignettes, mixed up and in random order, nearly identical results were obtained. Another limitation was that the sono-rheumatologist was not completely blinded and received summary information about patients before MSUS, which could have influenced his judgment.

The strength of this study was the idea of using a probabilistic approach to determine if MSUS improved the diagnostic certainty on a five-point scale, which indeed it did. A further strength of this study was that MSUS could influence the rheumatologic judgment during the investigation and finally the diagnosis. As Ceponis *et al*. showed previously, MSUS of the hand and wrist can increase the physicians’ confidence in their clinical decisions [13] and we believe that our MSUS results could also influence rheumatologists in their decisions to make a diagnosis or to re-refer the patients. We established that our rheumatologists became more certain of the presence or absence of inflammatory arthritis when they knew the MSUS findings. In other words, we showed that the rheumatologists were influenced by the ultrasound findings. Furthermore, they performed well after being influenced. Ultrasound helped them indentify with certainty more patients with and without inflammatory arthritis, and most of the patients with positive MSUS finding had anti-rheumatic treatment (mostly DMARDs) at the end of the follow-up time. Additionally, the utility of MSUS was most impressive when diagnostic uncertainty was maximal, as might have been anticipated. This is the group where a decision to prescribe anti-rheumatic therapy is more difficult and where ultrasound can be more useful by increasing diagnostic certainty on the presence/absence of inflammatory arthritis. When the diagnosis became more certain towards the positive end then the impact of MSUS became somewhat less although we still observed a numeric increase in the use of anti-rheumatic treatment.

## Conclusion

MSUS, when added to routine clinical and laboratory examinations, greatly increased the diagnostic certainty in patients referred for the evaluation of inflammatory arthritis. Moreover, inflammatory arthritis in general and RA specifically were diagnosed with higher certainty based on MSUS findings.

## Abbreviations

ACPA: anti citrulinated protein antibody
ACR: American College of Rheumatology
CDUS: color Doppler ultrasound
CRF: case-report form
DMARD: disease-modifying anti-rheumatic drug
EULAR: European League against Rheumatism
MCP: metacarpophalangeal
MHz: mega Hertz
MSUS: musculoskeletal ultrasound
MTP: metatarsophalangeal
PIP: proximal interphalangeal
RA: rheumatoid arthritis
RF: Rheumatoid factor
SRQ: Swedish Rheumatology Quality registry
UIA: Undifferentiated inflammatory arthritis
